# MicroRNA-362 negatively and positively regulates SMAD4 expression in TGF-β/SMAD signaling to suppress cell migration and invasion

**DOI:** 10.7150/ijms.50871

**Published:** 2021-02-18

**Authors:** Han Ping Cheng, Chiu-Jung Huang, Ming-Long Tsai, Hooi Tin Ong, Soon Keng Cheong, Kong Bung Choo, Shih-Hwa Chiou

**Affiliations:** 1 Centre for Stem Cell Research, Faculty of Medicine and Health Sciences, Universiti Tunku Abdul Rahman, Selangor, Malaysia.; 2 Postgraduate Program, Faculty of Medicine and Health Sciences, Universiti Tunku Abdul Rahman, Selangor, Malaysia.; 3 Department of Preclinical Sciences, Faculty of Medicine and Health Sciences, Universiti Tunku Abdul Rahman, Selangor, Malaysia.; 4 Dean's Office, Faculty of Medicine and Health Sciences, Universiti Tunku Abdul Rahman, Selangor, Malaysia.; 5 Department of Medical Research, Taipei Veterans General Hospital, Taipei, Taiwan.; 6 Department of Animal Science & Graduate Institute of Biotechnology, Chinese Culture University, Taipei, Taiwan.; 7 Institutes of Pharmacology, National Yang-Ming University, Taipei, Taiwan.; 8 Institute of Clinical Medicine, National Yang-Ming University, Taipei, Taiwan.

**Keywords:** microRNA-362, EMT/MET, SMAD4, TGF-β/SMAD signaling pathway, cell migration/invasion, metastasis.

## Abstract

Cell migration and invasion are modulated by epithelial-to-mesenchymal transition (EMT) and the reverse MET process. Despite the detection of microRNA-362 (miR-362, both the miR-362-5p and -3p species) in cancers, none of the identified miR-362 targets is a mesenchymal or epithelial factor to link miR-362 with EMT/MET and metastasis. Focusing on the TGF-β/SMAD signaling pathway in this work, luciferase assays and western blot data showed that miR-362 targeted and negatively regulated expression of SMAD4 and E-cadherin, but not SNAI1, which is regulated by SMAD4. However, miR-362 knockdown also down-regulated SMAD4 and SNAI1, but up-regulated E-cadherin expression. Wound-healing and transwell assays further showed that miR-362 knockdown suppressed cell migration and invasion, effects which were reversed by over-expressing SMAD4 or SNAI1, or by knocking down E-cadherin in the miR-362 knockdown cells. In orthotopic mice, miR-362 knockdown inhibited metastasis, and displayed the same SMAD4 and E-cadherin expression profiles in the tumors as in the *in vitro* studies. A scheme is proposed to integrate miR-362 negative regulation via SMAD4, and to explain miR-362 positive regulation of SMAD4 via miR-362 targeting of known SMAD4 suppressors, BRK and DACH1, which would have resulted in SMAD4 depletion and annulment of subsequent involvement in TGF-β signaling actions. Hence, miR-362 both negatively and positively regulates SMAD4 expression in TGF-β/SMAD signaling pathway to suppress cell motility and invasiveness and metastasis, and may explain the reported clinical association of anti-miR-362 with suppressed metastasis in various cancers. MiR-362 knockdown in miR-362-positive cancer cells may be used as a therapeutic strategy to suppress metastasis.

## Introduction

Cancer cells of malignant tumors metastasize to distant sites to form secondary tumor growths. To gain motility, epithelial cells first lose cell polarity and cell-cell adhesion to become mesenchymal stem cells in the epithelial-to-mesenchymal transition (EMT) process, defined by expression of a panel of mesenchymal markers, typically SNAI1, and suppression of epithelial markers typically represented by the adhesion molecule E-cadherin (*CDH1*) (reviewed in 1). On the other hand, the reverse mesenchymal-to-epithelial transition (MET) is required for the metastasized cells to re-acquire epithelial phenotype for establishment at the secondary sites (reviewed in 2). Previous studies have shown that microRNAs (miRNAs), which are negative regulators of gene expression, play important roles in cancer metastasis (reviewed in 3) through modulation of the EMT and MET processes 4,5. Dynamic transition between EMT and MET has also been associated with cancer cell stemness 6. In a previous study in our laboratory, miR-362-5p was identified as one of top 10 miRNAs down-regulated in Yamanaka factors-reprogrammed colorectal cancer cells 7. Both the miR-362-3p and -5p species were further predicted to be involved in regulating expression of the EMT- and MET-associated genes, possibly via the phosphatidylinositol-3-kinases-protein kinase B (PI3K-Akt) and transforming growth factor-beta (TGF-β) signaling pathways. In separate studies, miR-362 has been reported to enhance cell migration and invasion in various cancers 8-16 and in non-cancer cells, including trophoblastic cells under hypoxia and atherosclerotic vascular smooth muscle cells 17,18. Hence, miR-362 clearly plays a regulatory role in the EMT and MET processes in driving cancer cell metastasis. Surprisingly, none of the miR-362 targets identified in these studies included any of the well-characterized EMT- and MET-associated factors 8,12,13,16,19-21.

TGF-β is an important driver of cancer progression by promoting EMT in tumor cells to expedite cancer-cell stemness, metastasis and invasion (reviewed in 22,23). The TGF-β transduction signals act via the central modulator SMAD to mediate transcription regulation of a cascade of downstream genes in the TGF-β/SMAD signaling pathway 9,24,25. One of the immediate targets of SMAD4 activation is the transcription factor Snail family transcriptional repressor 1, SNAI1, which, in turn, inhibits expression of the epithelial marker E-cadherin downstream of the TGF-β/SMAD signaling pathway. The present study aimed to determine if miR-362 targeted and regulated expression of SMAD4 and the TGF-β/SMAD signaling pathway to modulate migration and invasion of cancer cells. The results, indeed, revealed that miR-362 drives the TGF-β/SMAD signaling pathway via regulating expression of SMAD4 in modulating cellular migration and invasion, superseding the various reported yet not validated miR-362 target proteins.

## Materials and Methods

### Cell lines

Human breast cancer MCF7 (ATCC^®^ HTB22™) and colorectal adenocarcinoma HCT-15 (ATCC^®^ CCL-225™) cell lines were purchased from the American Type Culture Collection (Manassas, VA, USA), and were cultured in RPMI‑1640 and DMEM high-glucose medium (Thermo Fisher Scientific, Inc., MA, USA), respectively, supplemented with 10% fetal bovine serum (FBS) (Gibco, MD, USA) in a humidified atmosphere at 37 °C with 5% CO_2_.

### Animal studies

Immunodeficient BALB/c nude mice (male, 6-8 weeks old) were obtained from National Laboratory Animal Center, Taipei. All mice were bred and maintained under specific pathogen-free conditions with a 12-hour light/dark cycle, with temperature of the facility maintained between 20 - 22 °C. The mice were fed commercial diet and given water *ad libitum*. All experimental procedures involving mice were approved by the Institutional Animal Care and Use Committee (IACUC) of Taipei Veterans General Hospital, Taipei and in compliance with the guidelines on animal welfare (reference number: 2018-026). At the end of experiment, mice used in this study were sacrificed by cervical dislocation.

### Transient transfection of miRNA mimics

MirVana hsa-miR-362-3p or -5p mimics (cat. no. 4464066) and negative control #1 (NC) (cat. no. 4464058) were synthesized by Ambion (CA, USA). The synthetic miRNA mimics were transfected into MCF7 cells by using Lipofectamine 2000 Reagent (Invitrogen, MA, USA) according to the manufacturer's protocol. MCF7 was used in the miRNA overexpression experiments because of low endogenous miR-362 levels. The day before transfection, the MCF7 cells were seeded at 7.5 x 10^5^ cells/well in a 6-well plate. The cells were trypsinized for further analysis after incubation for 48 h.

### Transduction of lentiviral miRNA sponge constructs for miR-362 knockdown

Three lentiviral constructs containing miRNA sponges, namely pLKOAS7w.mCherry-CMV-d2EGFP-miR362-3p or -5p, or CXCR, a negative control vector, were obtained from the National RNAi Core Facility, Academic Sinica, Taipei, Taiwan. The miRNA sponge constructs were individually transfected into the packaging cell line 293FT cells by using the TransIT-LT1 transfection reagent diluted (Mirus Bio, WI, USA). Virus supernatant was harvested after 48 h and filtered through a 0.45-µm pore size PVDF filter. For transduction, the virus supernatant supplemented with 8 µg/ml polybrene (Merck Millipore, Darmstadt, Germany) was added to the HCT-15 cells seeded at 5.0 x 10^5^ cells/well. HCT-15 was used in miRNA knockdown on the basis that the cells were highly permissive for lentiviral vector transduction, and also for robust post-transduction growth. Medium was refreshed with fresh complete medium supplemented with 4 μg puromycin 48 h post-transduction. Growth medium, supplemented with puromycin, was replaced every two days for selection of the transduced cells. The transduced HCT-15 cells were checked for green fluorescence protein (GFP) and Mcherry fluorescence signals under fluorescent light at 490 and 540 nm, respectively. Puromycin-selected HCT-15 cells stably expressing the miRNA sponges were expanded and harvested for quantitative miRNA analysis using the Taqman miRNA assays to ensure miRNA knockdown before being used for further analysis.

### Protein lysate preparation and western blot analysis

Protein lysates were prepared from cells using 1X RIPA lysis buffer (Merck) and western blot analysis was performed as described 7. The membranes were probed with primary antibodies overnight at 4 °C. Antibodies against SMAD4 (at 1:500 dilution) (cat. no. 3854), SNAI1 (1:250) (cat. no. 3879), E-cadherin (CDH1) (1:1,000) (cat. no. 3195) and GAPDH (1:2,000) (cat. no. 5174) were obtained from Cell Signaling Technology, MA, USA. The proteins were detected using a horseradish peroxidase-conjugated secondary antibody (1: 5,000, Abcam, Cambridge, UK) (cat. no. ab205718) incubating for 1 h at room temperature. Protein bands were visualized with the Amersham ECL western blotting substrate (GE Healthcare, PA, USA), according to the manufacturer's protocol.

### Plasmid construction and luciferase assays

PCR products containing the predicted miR-362-3p and/or -5p binding sites were amplified using specific primers (Suppl. Table S1) and were cloned into the 3'-end of the firefly luciferase gene in the dual reporter vector pmirGLO (Promega, WI, USA). To generated luciferase mutants, site-directed mutagenesis of the miRNA seed sequences of the miR-362 target sites of the transcript was performed using the standard overlap-extension protocol using specific primers for PCR amplification (Suppl. Table S1). The *CDH1* double mutant was obtained by sequential mutagenesis and cloning. For luciferase assays, co-transfection of the luciferase constructs and a miR-362-3p or -5p mimic, or a negative control mimic (Ambion, TX, USA) into MCF7 cells was performed in triplicates by using Lipofectamine™ 2000 (Invitrogen) according to the manufacture's protocol. Luciferase assays were performed in triplicates 48 h post-transfection using the Dual-Luciferase Reporter 1000 Assay kit (Promega). The data presented were obtained from three independent experiments.

### Wound Healing Assay

MiR-362-3p or -5p mimic- or NC-transfected MCF7 cells (see above) were seeded at 3 ×10^5^ cells/mL in each of the two chambers of a culture-insert dish (Ibidi, Martinsried, Germany) to create a 500-μm cell-free gap by exclusion. At 24 h post-seeding, the insert was gently removed and the cells were washed once with 1X PBS before overlaid with warm complete RPMI medium. Images were taken at 0 and 24 h to monitor wound closure. The extent of wound closure was calculated by performing an 8-bit image analysis and MRI Wound Healing Tool plugin provided by the ImageJ software (NIH, USA). The percentage of a wound area after 24 h was calculated relative to the original area at 0 h for each experimental condition.

### Transwell migration and invasion Assays

For migration assays, 2.0 x 10^5^ cells/mL MCF7 cells were first seeded onto the top chamber of an 8.0-μm FluoroBlok transwell insert (BD, NJ, USA). RPMI medium supplemented with 10% FBS was added to the bottom chamber and incubated overnight at 37 °C with 5% CO_2._ For the invasion assays, FluoroBlok Cell Culture Inserts were coated with Geltrex LDEV-Free Reduced Growth Factor Basement Membrane Matrix (Gibco, cat. no. A1413202). For seeding on top of the coated wells, 2.0 x10^5^ cells/mL HCT-15 cells were used. After 24 h of incubation, cells that had migrated to or invaded the bottom side of the insert were fixed and stained with propidium iodide, and were counted under a fluorescence microscope. Five images of randomly selected fields were captured on each filter using the CellSens software for microscopic image analysis.

### Protein gene over-expression and siRNA knockdown

For protein gene over-expression, the coding sequences of the genes were PCR-amplified and cloned into the p3xFLAG-Myc-CMV™-26 expression vector (Sigma). The cloned gene inserts were verified by sequencing. For gene expression knockdown, specific Dicer-Substrate siRNA (DsiRNA) were purchased from (IDT Technologies, Iowa). SiRNA duplexes (Trifecta kit; IDT Technologies, cat. no. 102198208) were mixed with lipofectamine RNAiMAX reagent (Invitrogen) according to the manufacturer's instructions. Mock transfection with a negative control (NC) non-targeting siRNA (IDT Technologies, cat. no. 51-01-14-03) was used as a control. At 48 h post-transfection, cells were harvested and protein lysates were prepared to confirm knockdown by western blot analysis before further use.

### Orthotopic and ectopic mouse models

To generate orthotopic mice, HCT-15 cells stably knockdown of miR-362-3p or -5p by the miRNA sponges, or control cells, were harvested by trypsinization and transferred to a serum-free medium. Nude mice were anesthetized by an intraperitoneal injection of pentobarbital sodium (1.5 mg per 20 g bodyweight). After sterilization, a small abdominal incision was made, and the cecum was exteriorized. The miR-362 knockdown cells (2.0 × 10^6^) in 20 μL serum-free medium prepared above were inoculated onto the cecal walls using 30-gauge needles. The cecum was returned to the peritoneal cavity and the abdominal wound was closed. For each miRNA sponge, six mice were injected using three different cell batches, two mice per cell batch. Mice that survived up to 80 days were sacrificed for examination of tumor formation and metastasis under white or fluorescence light. To generate ectopic mice, approximately 5 × 10^6^ viable HCT-15 cells stably transduced with miR-362-3p or -5p sponges were injected subcutaneously into the dorsal flank of a BALB/c nude mouse, whereas the negative control-transduced cells were injected into the other flank of the same mouse. Eighteen days post inoculation, the mice were sacrificed and the tumors were harvested for protein lysate preparation for western blots and in immunohistochemistry analysis.

### Statistical analysis

Data were analyzed and presented as means ± SEM of three independent experiments done in triplicates. The differences between treated and control groups were analyzed by paired Student's *t*-test (two-tailed distribution). *p-*value ≤ 0.05 was considered as statistically significant.

## Results

### MiR-362 regulates expression of SMAD4 and E-cadherin of the TGF-β/SMAD signaling pathway

**Figure 1:**
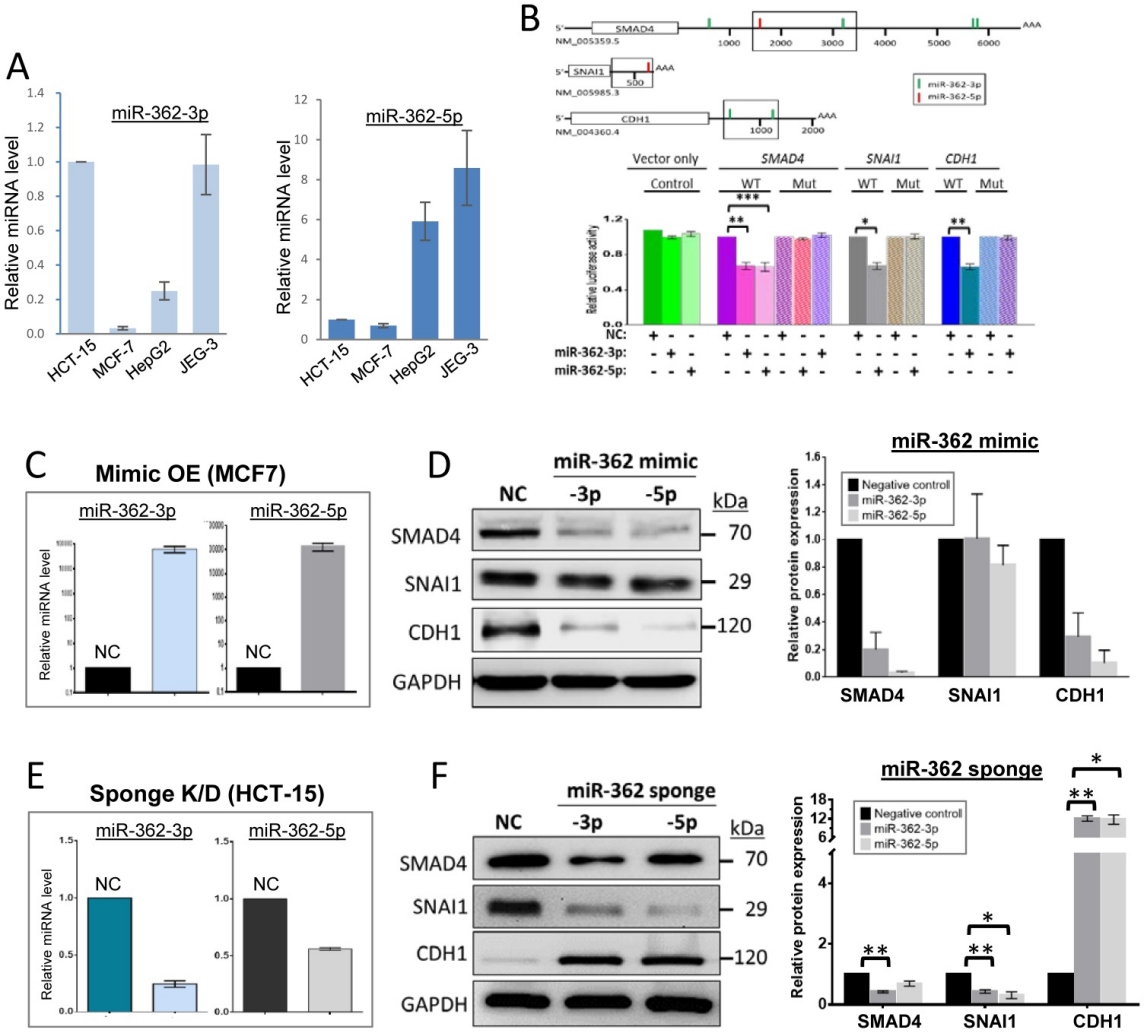
MiR-362 regulates expression of SMAD4 and E-cadherin of the TGF-β/SMAD signaling pathway. (A) Relative expression levels of miR-362 in cancer cell lines determined by qRT-PCR. (B) Predicted miR-362 targeting of the SMAD4, SNAI1 and CDH1 transcripts. Top panel: Predicted miR-362-3p (vertical green bars) and -5p (red bars) target sites in the 3'-untranslated regions (3'UTR) of the transcripts. The boxed 3'UTR segments were cloned into the pmirGLO vector for luciferase analysis. The GenBank accession numbers of the transcript sequences are also shown. AAA, polyA tail. The scale bars (in bp) indicating nucleotide positions are only for the 3'UTR sequences. Bottom panel**:** Validation of miR-362 targeting determined by luciferase assays. In each case, a wild-type (WT) or seed sequence-mutated (Mut) luciferase construct was co-transfected into MCF7 cells in triplicates in the presence (“+”) or absence (“-'') of a miR-362-5p or -3p mimic, or a sequence-scrambled negative control (NC) mimic, before performing the dual luciferase assays. The fold change in relative luciferase activity was plotted. The mean ± SEM data shown were obtained from three independent experiments. (C, D) MiR-362 over-expression (C) down-regulated expression of SMAD4 and E-cadherin but not SNAI1 (D). MiR-362 over-expression (OE) was achieved by transient transfection of a miR-362-3p or -5p mimic in MCF7 cells; NC was a scrambled oligonucleotide. (E, F) MiRNA-362-knockdown (E) led to E-cadherin over-expression but suppressed SMAD4 and SNAI1 expression (F). MiR-362 knockdown (KD) was achieved by transduction of a miR-362-3p or -5p sponge vector in HCT-15 cells; NC was a construct with a scrambled sequence insertion. In (D) and (F), quantification of the western blot data (mean ± SEM) from three independent experiments is shown in the right panels of the blots. In all subfigures, *p < 0.05, **p < 0.01 and ***p < 0.001 relative to the negative controls (NC).

For brevity in this work, miR-362-3p and -5p are collectively designated as miR-362, or stated otherwise. As miR-362 has been clinically reported to be present in a number of cancers, cancer cell lines were first tested for the presence of miR-362. The results confirmed detection of miR-362 in different cancer cell lines, and showed that the colorectal cancer (CRC) cell line HCT-15, the hepatocellular cancer cell line HepG2 and the choriocarcinoma cell line JEG-3 showed the highest miR-362 levels whereas the breast cancer cell line MCF7 was low in both the miR-362-3p and -5p levels (Fig. 1A).

To first determine if miR-362 targets selected factors of the TGF-β/SMAD signaling pathway affecting EMT, the 3'untranslated regions (3'UTR) of the transcripts of *SMAD4* and the downstream *SNAI1* and E-cadherin 1 (*CDH1*) were first probed for miR-362-targeted sites. Both miR-362-3p and -5p target sites were identified in the *SMAD4* transcript; two miR-362-3p sites were identified in the *CDH1* transcript whereas there was only one -5p site found in the short 3'UTR of *SNAI1* (Fig. 1B, top panel). The 3'UTR segments harboring the identified miR-362 target sites were cloned into the luciferase vector, pmirGLO, and the luciferase constructs were transfected into the low miR-362-level MCF7 cells but in the presence of a miR-362-3p or -5p mimic for luciferase assays. Significant reduction of luciferase activities was observed for all the constructs relative to control cells transfected with a negative control (NC) miRNA mimic (Fig. 1B, bottom panel, WT bars), predicting miR-362 targeting. When the seed sequences of the miR-362 target sites of the luciferase constructs of the three genes were mutated, co-transfection of the miR-362-3p or -5p mimic did not elicit luciferase activity suppression (Fig. 1B, bottom panels, mutant bars), consistent with miR-362 targeting of the *SMAD4*, *SNAI1* and *CDH1* transcripts.

To confirm miR-362 negative regulation, a 15-fold miR-362 over-expression (OE) was first achieved by transient transfection of a miR-362-3p or -5p mimic into MCF7 cells, which showed low endogenous miR-362 levels as described above (Fig. 1C). Western blot results showed that miR-362 over-expression led to significant down-regulated protein levels of SMAD4 and E-cadherin but not SNAI1 (Fig. 1D). The SMAD4 and E-cadherin results were consistent with the luciferase data prediction, and the unaffected SNAI1 results were consistent with the absence of a miR-362-3p target site in the 3'UTR, and that the -5p site was not directly targeted despite the luciferase assay data prediction (Fig. 1B). It is important to note here that it has been well established that SNAI1 is under direct regulation by the SMAD4/phospohrylated SMAD1/2 complex 9, 24, 25. Hence, the SNAI1 results presented in this work were likely a consequence of changes in the expression levels of the SMAD4 regulator.

To obtain further evidences to support the miR-362 over-expression data, effects of miR-362 knockdown (KD) on protein levels were also analyzed. MiR-362 knockdown was achieved by the use of a miR-362-3p or -5p lentiviral-sponge vector (Fig. 1E). For this purpose, HCT-15 cells were used for viral-vector permissiveness and for growth robustness in the selection of stable cell clones after transfection (see Materials and Methods). In the miR-362 sponge-treated cells, miR-362-3p and -5p were significantly down-regulated by ~75% and ~50%, respectively, relative to the negative-control sponge-transfected cells (Fig. 1E). Surprisingly, miR-362 knockdown also appeared to down-regulate the SMAD4 levels, abeilt with a smaller effects of suppression of about 2-fold change (Fig. 1F) whereas miR-362 over-expression resulted in a 5- to 20-fold down-regulated SMAD4 levels relative to the control (Fig. 1F). The data collectively suggest that miR-362 acts predominatly as a negative regulator when over-expressed. On the other hand, miR-362 could also act as a positive regulator possibly as a results of involvement of miR-362-targeted SMAD4 suppressors, or miR-362 feedback- or feedforward regulatory loops when other miR-362-modulated proteins upstream outside the TGF-β/SMAD signaling pathway are recruited (26,27; see Discussion below). The SNAI1 level was also down-regulated 2- to 3-fold on miR-362 knockdown, which could be attributed to miR-362 knockdown-induced down-regulation of SMAD4 as described above 24. On the other hand, miR-362 knockdown resulted in up-regulated E-cadherin levels (Fig. 1F), echoing miR-362 over-expression-induced negative regulation shown above (Fig. 1D), indicating that E-cadherin is under direct miR-362 regulation. Taken together, the data indicate that SMAD4 and E-cadherin, but not SNAI1, are under miR-362 regulation.

### Mir-362 promotes cell migration and invasion* in vitro*

**Figure 2:**
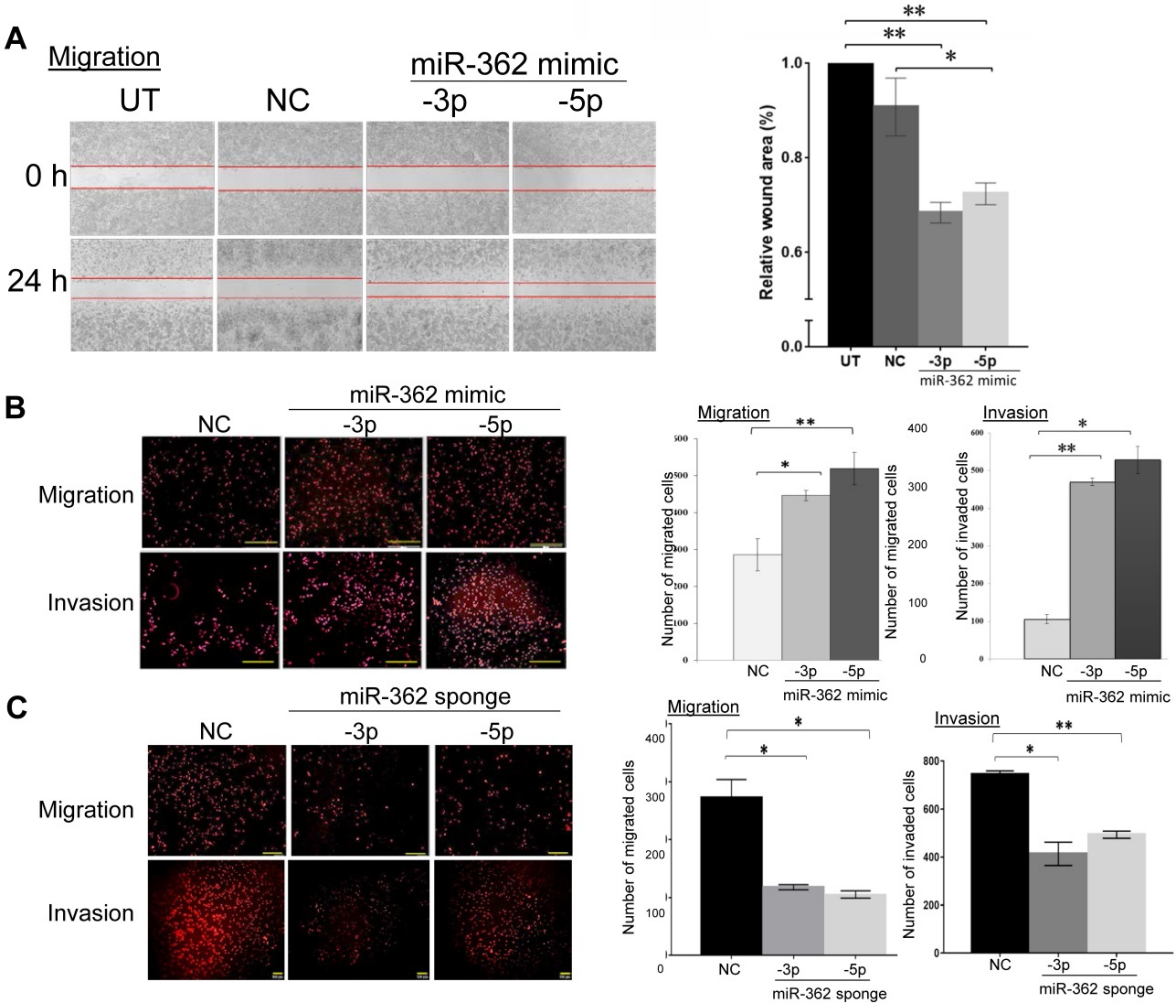
MiR-362 promotes cell migration and invasion *in vitro*. (A) Wound‑healing assays of MCF7 cells untransfected (UT) or transfected with a miR‑362‑5p or -3p, or a negative control (NC) mimic. The images were captured before (0 h) and 24 h after wound healing began. Quantification of the data obtained from three independent experiments is shown in the bar charts on the right, normalizing to data of the untransfected cells. (B, C) Effects of miR-362 over-expression (B) or knockdown (C) on cell migration and invasion in transwell assays. MiR-362 over-expression and knockdown were achieved as described in Fig. 1C above. Representative images captured 24 h post-transfection (left panels) and the mean ± SEM from three independent experiments relative to the negative control groups (right panels) are shown; *p< 0.05, **p< 0.01 relative to the negative controls (NC).

MiR-362 involvement in cell migration and invasion was next investigated. In wound-healing assays in MCF7 cells, miR-362 mimic transfection significantly enhanced cell migration, as indicated by a ~1.5-fold significant reduction of the wound area relative to the negative control (NC) mimic-transfected cells (Fig. 2A). Transwell migration assays also demonstrated that miR-362 over-expression resulted in 1.7- to 2-fold significant increases in the number of migrated cells into the transwells (Fig. 2B), and that the number of invading miR-362 mimic-transfected MCF7 cells was also significantly increased by >3-fold when compared to the NC-transfected cells (Fig. 2B). On the other hand, the reverse was observed in transwell assays when miR-362 was knocked down via miR-362 sponges in suppressing both cell migration and invasion (Fig. 2C). Collectively, the data show that miR-362 promotes both cell migration and invasion *in vitro*.

### SMAD4 and SNAI1 overexpression and E-cadherin knockdown restore the cell migration and invasion phenotypes in miR-362-knockdown cells

**Figure 3:**
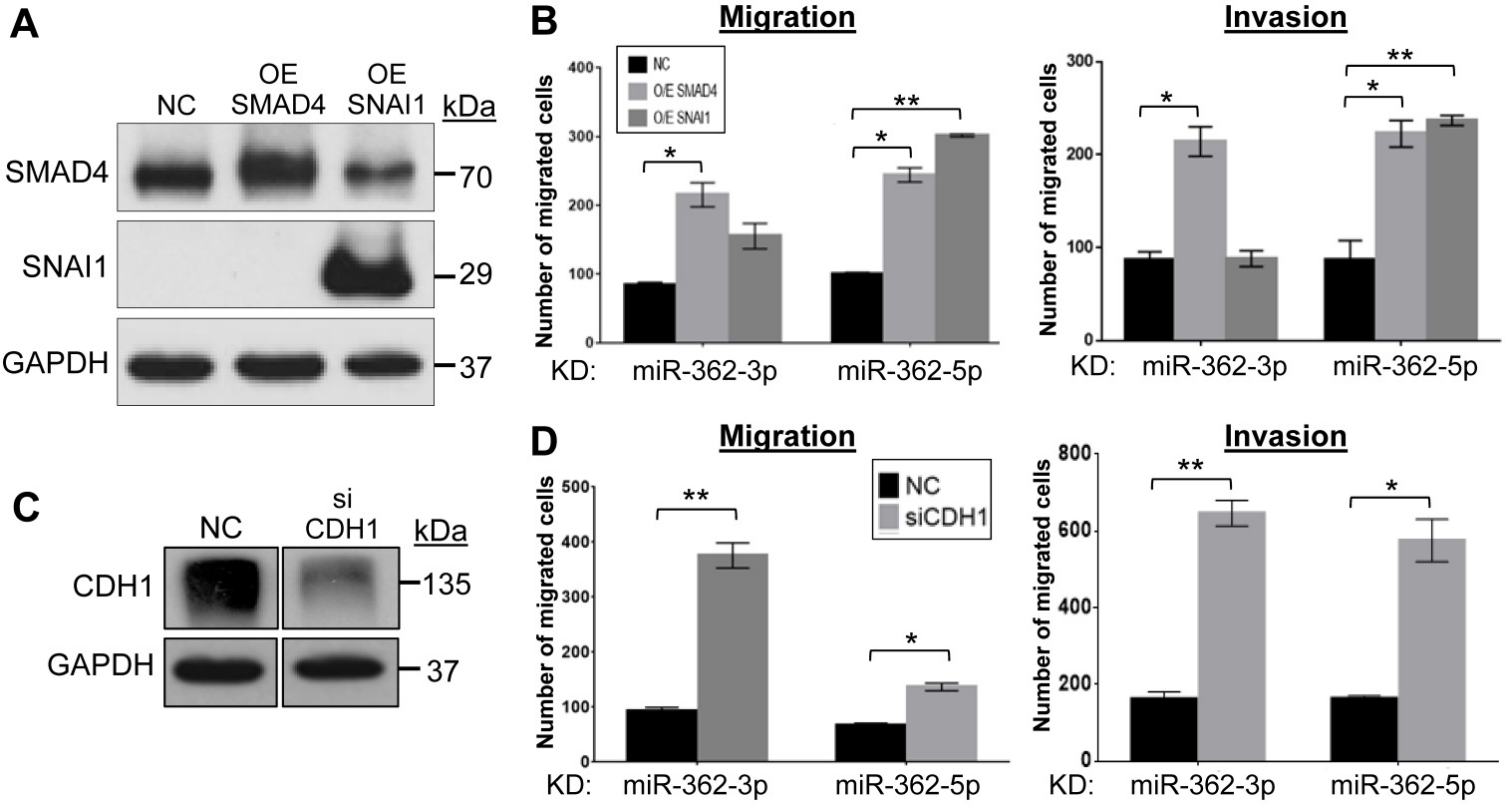
SMAD4 and SNAI1 overexpression and E-cadherin knockdown restore the cell migration and invasion phenotypes in miR-362-knockdown cells *in vitro*. (A, B) Effects of SMAD4 and SNAI1 over-expression on cell migration and invasion in the miR-362-knockdown HCT-15 cells. SMAD4 and SNAI1 over-expression (OE) was achieved by transient transfection of the respective expression plasmid constructs, followed by western blot analysis (A) and effects on cell migration and invasion were assayed in transwell assays (B). (C, D) Effects of E-cadherin knockdown on cell migration and invasion in the miR-362-knockdown HCT-15 cells. E-cadherin knockdown was achieved by transfection of a siCDH1 siRNA (C) and effects on migration and invasion (D) were assayed in transwell plates. In the over-expression experiments, a blank (NC) expression plasmid was used as a control; in the siRNA knockdown experiments, a validated negative control (NC) siRNA was used. Data of mean ± SEM from three independent experiments are shown. *p<0.05; **p<0.01 values were calculated relative to the negative control samples.

To more directly link the miR-362 knockdown-induced altered protein levels (Fig. 1F) and the suppressed cell migration and invasion phenotype observed (Fig. 2), the down-regulated SMAD4 and SNAI1 levels were restored by transfection of the respective expression plasmids in the miR-362-knockdown cells (Fig. 3A). In transwell assays, restoring SMAD4 expression resulted in enhanced migration and invasion activities of the miR-362-knockdown cells (Fig. 3B), alleviating the suppressed migration and invasion phenotype on miR-362 knockdown. However, SNAI1 over-expression only resulted in significantly augmented migration and invasion of the miR-362-5p-knockdown cells, but not the -3p cells, suggesting that the miR-362-5p regulation on SMAD4 may have more profound downstream and immediate regulatory effects than the miR-362-3p species on SNAI1. Conversely, the up-regulated E-cadherin levels in the miR-362 sponge cells were knocked down by an E-cadherin-specific siRNA (Fig. 3C). In transwell assays, siRNA knockdown of E-cadherin also restored the miR-362-induced suppressed migration and invasion abilities of the cells (Fig. 3D). Taken together, the data presented in Figures 1 to 3 indicate that miR-362 modulates cell migration and invasion via regulation of expression of SMAD4, SNAI1 and E-cadherin.

### MiR-362 knockdown suppresses metastasis *in vivo*

**Figure 4:**
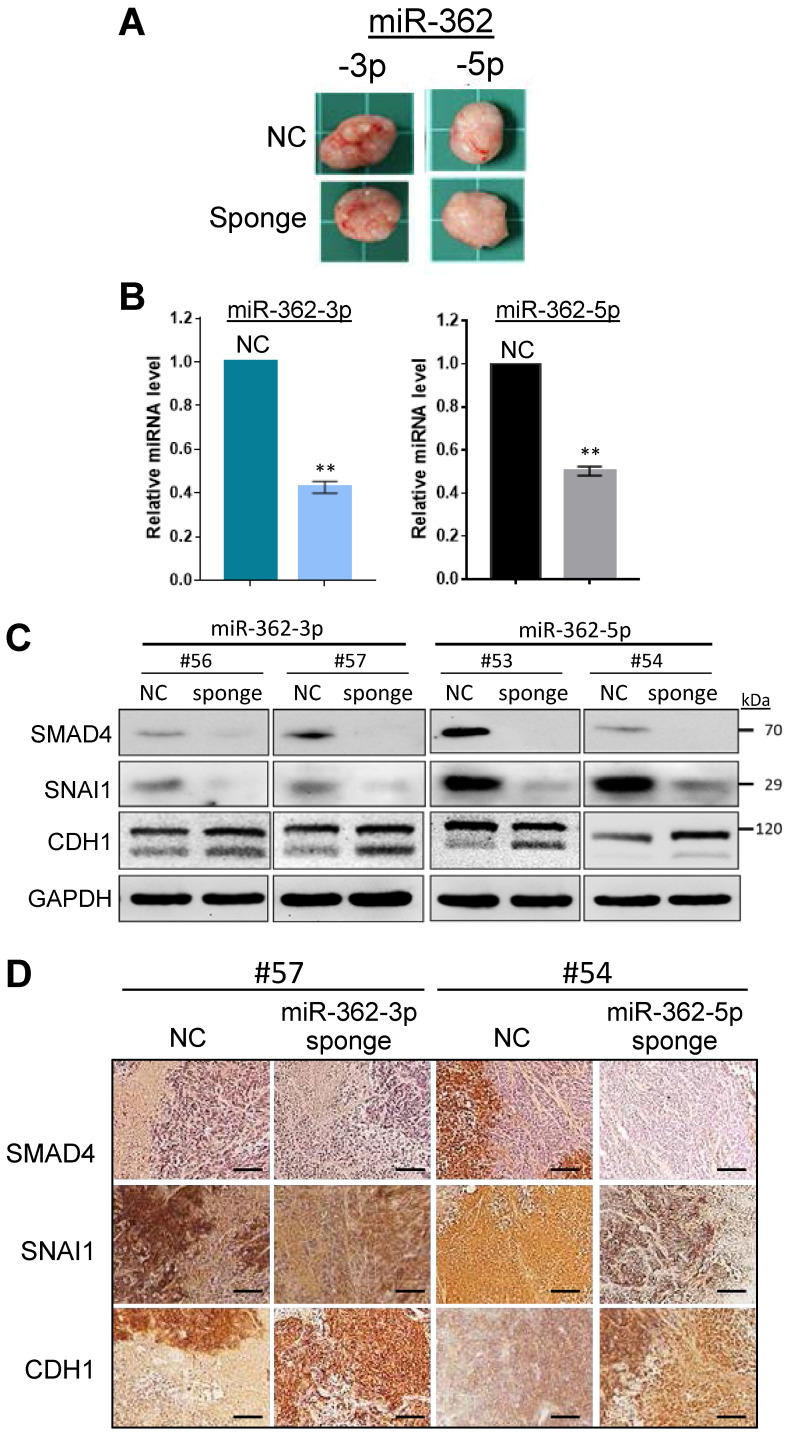
Similar *in vivo* expression patterns of SMAD4, SNAI1 and E-cadherin as in *in vitro* on miR-362 knockdown. (A) Tumors developed in ectopic mice generated by subcutaneous injection of miR-362-knockdown cells, or cells transduced with a negative control (NC) sponge vector. Total RNAs and protein lysates were prepared from the tumors for further analysis. (B) Knockdown miR-362 expression in the mouse tumors determined by qRT-PCR. Data were derived from analysis of three different tumors harvested from three independently generated mice using different cell batches (see Suppl. Fig. S1). (C, D) Protein expression in the tumors of the miR362-knockdown ectopic mice as shown in western blots (C) and immunohistochemical staining (D). In (D), scale bar: 20 μm.

Before investigating the effects of miR-362 on metastasis of tumor cells in *vivo*, the expression patterns of SMAD4, SNAI1 and E-cadherin *in vivo* were first examined in tumors developed in ectopic mice generated by subcutaneous injection of the sponge-induced miR-362-knockdown HCT-15 cells, or a negative control sponge. Mice were independently inoculated using three different cell batches and were designated different mouse numbers; tumor masses of the different mice were harvested on day 18 post-injection (Fig. 4A). Real-time RT-PCR analysis of RNA preparations of the tumors harvested from the three independently generated mice showed consistent results (Suppl. Fig. S1), which collectively indicated significantly lower miR-362 levels relative to the NC levels (Fig. 4B), indicating successful sponge-induced miR-362 suppression in the tumors as in the cell lines, which were used to generate the tumors, described above (Fig. 1E). The protein lysates of the tumors were subjected to western blot (Fig. 4C) and immunohistochemical analyses (Fig. 4D). The results consistently showed down-regulated SMAD4 and SNAI1 and up-regulated E-cadherin levels in miR-362 knockdown tumor samples, consistent with the *in vitro* results.

**Figure 5:**
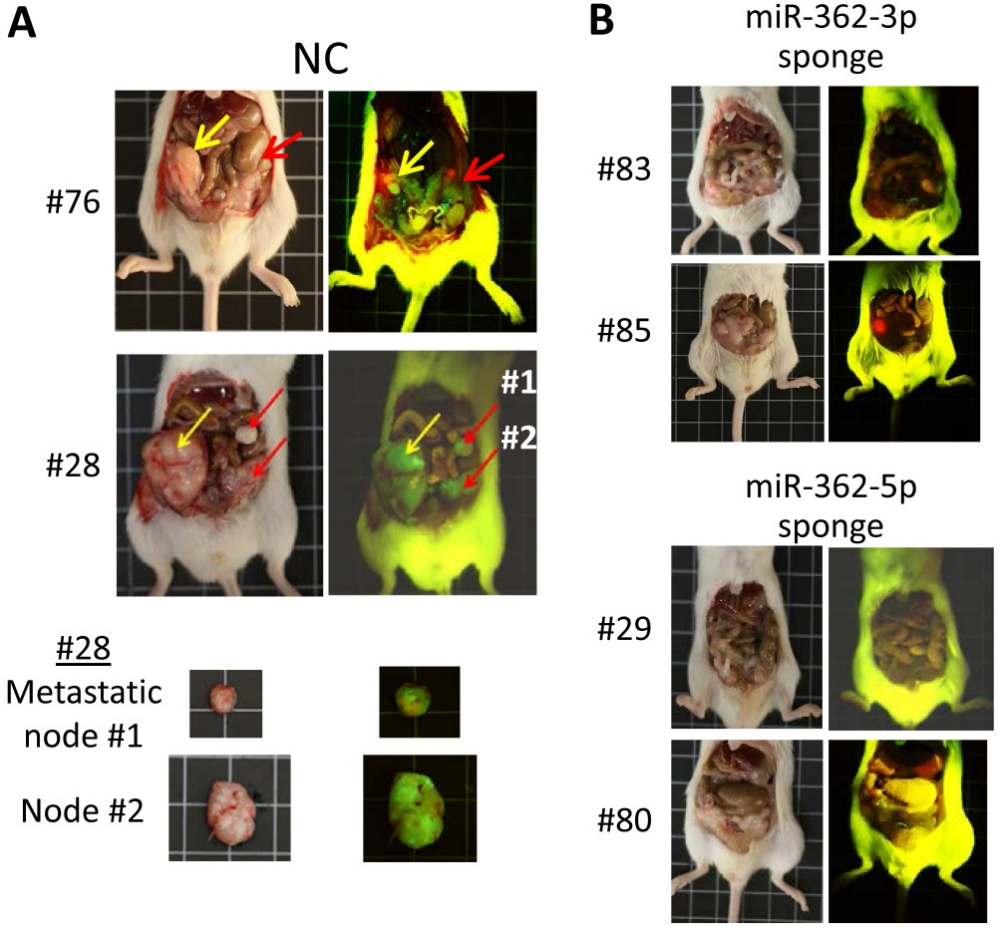
MiR-362 knockdown suppresses metastasis *in vivo*. (A) Tumor growth and metastasis of untreated HCT-15 cells (NC, negative control) in an orthotopic mouse model generated as described in Materials and Methods (Table 1). Images of tumors derived from two independently generated mice are shown; two metastatic nodes of mice #28 are also shown. In the images, yellow arrows indicate the initial tumor masses at the site of injection whereas red arrows indicate the metastasized tumor loci. (B) Suppressed tumor growth and metastasis of miR362-knockdown HCT-15 cells in orthotopic mice (see also Table 1). In each experimental group, images on the left and right were obtained under white or fluorescent light, respectively.

To assess miR-362-induced metastasis, orthotopic mice, which provide a metastasis-permissive microenvironment 28, 29, were generated by injecting miR-362 sponge-transfected HCT-15 colorectal cancer cells into the wall of the cecum, located at the start of the large intestine, of immune-deficient BALB/c nude mice. As in the ectopic mice, three different cell batches were used to generate mice with different designated numbers (Table 1). All the six mice injected with the NC sponge cells, i.e. without miR-362 knockdown, developed noticeable tumors within 3 weeks post-injection. The four mice that survived up to day 80 were sacrificed for further analysis. Three of the four NC mice showed 2-3 metastatic loci each (Fig. 5A & Table 1). In these negative control mice, the tumors showed green fluorescence, which indicated unsuppressed expression of the green fluorescence protein (GFP) marker gene of the miRNA sponge vector (Fig. 5A). Furthermore, multiple metastatic loci were evident as also indicated by the green fluorescence under fluorescent light (Fig. 5A, red arrows). Hence, the results indicated cancer cell metastasis in miR-362-positive orthotopic mice, consistent with the *in vitro* data that miR-362 over-expression enhanced cell migration and invasion. On the other hand, the four surviving mice injected with the miR-362-knockdown HCT-15 cells developed only minute tumors <50 mm^3^ in size, or no tumor, and that metastatic nodes were undiscernible in all the mice (Fig. 5B & Table 1), in agreement with the *in vitro* data that miR-362 knockdown suppressed cell migration and invasion. Taken together, the data showed that anti-miR-362 effectively suppresses metastasis *in vivo* via miR-362 down-regulation of SMAD4, and, therefore, SNAI1 via SMAD modulation, and up-regulation of E-cadherin in the TGF-β/SMAD signaling pathway.

## Discussion

**Figure 6:**
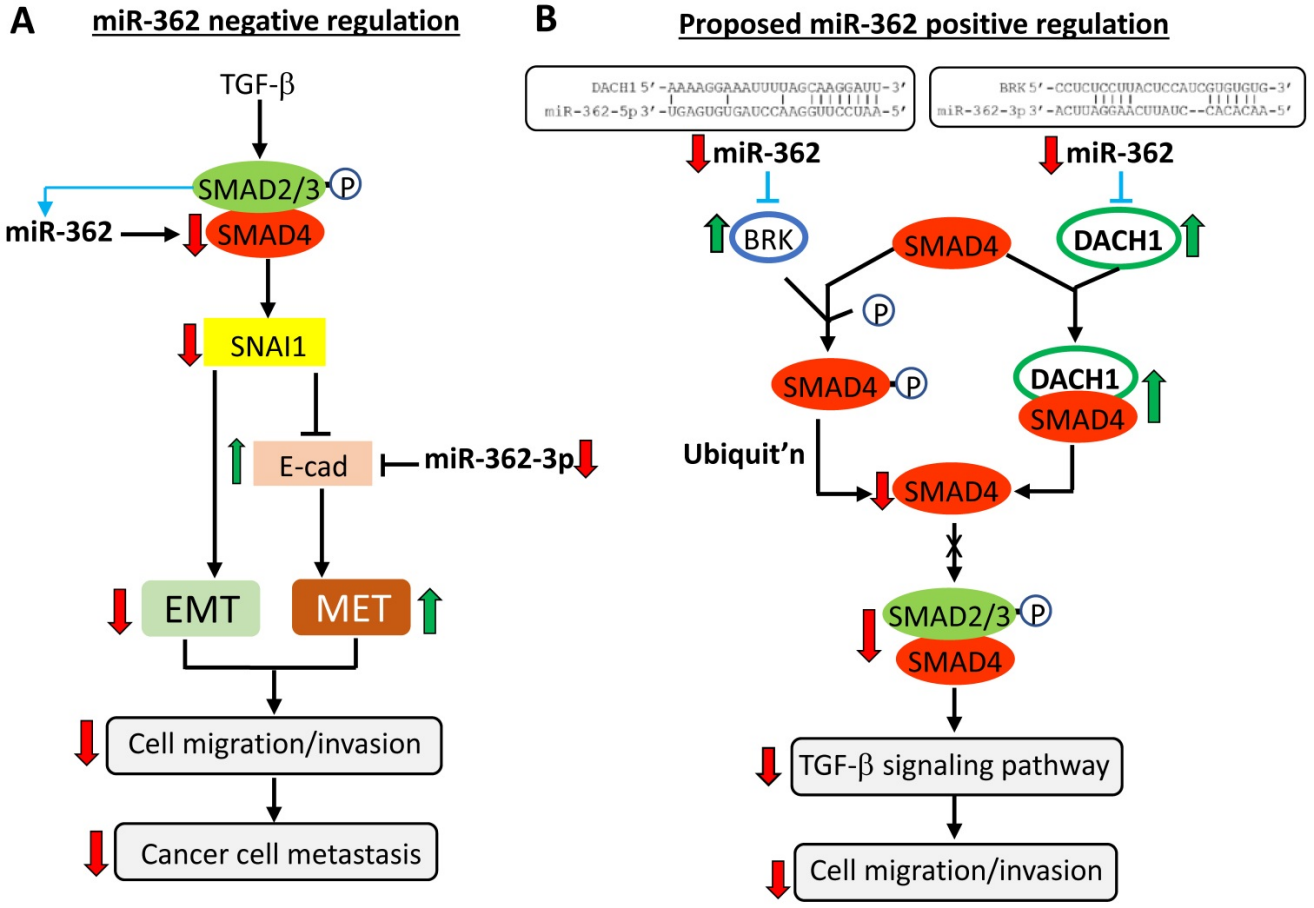
MiR-362-driven TGF-β/SMAD signaling modulation of cell migration and invasion: a proposed scheme. The scheme depicts the normal miR-362 negative regulation supported by data described in this work (A), and also a proposed mechanism of positive regulation of SMAD4 (B) in modulating the TGF-β signaling pathway and down-regulated expression of the EMT gene, *SNAI1*, and concurrently up-regulated expression of the MET gene, E-cadherin, leading to suppressed cell migration and invasion *in vitro* and cancer cell metastasis *in vivo*. See Discussion section for a full description of the scheme. Up- (green) and downward (red) pointing arrows indicate up- and down-regulation, respectively. Solid-line arrows and blunt-ends indicate activation and suppression, respectively, of the indicated events. The circled P denotes phosphorylation. Blue lines and arrows indicate predicted miR-362 targeting not further investigated in this work. In (B), the predicted *BRK* mRNA-miR-362-3p alignment shown at the top was derived by MicroT-CDS and that of *DACH1* mRNA-miR-362-5p by Targetscan and MicroT4-CDS (see also Suppl. Figure S2 for further details).

Expression of miR-362 has been shown to be clinically associated with a number of cancers, and that down-regulating miR-362 expression in miR-362-positive cancer cells is associated with suppressed tumor growth and metastasis 8, 12, 13, 16, 19-21. However, none of the reported miR-362 targets in these studies is a known mesenchymal or epithelial factor, or has been experimentally shown to modulate cell motility and invasiveness as we are reporting here. In the TGF-β/SMAD signaling pathway under normal circumstances, TGF-β ligands induce phosphorylation of SMAD2/3; the subsequently formed phosphorylated-SMAD2/3-SMAD4 complex enters the nucleus to up-regulate *SNAI1* expression and promotes EMT and cell migration and invasion 25, 30. SNAI1, in turn, inhibits E-cadherin expression to suppress the MET process, complementing the EMT action (Fig. 6A). This work showed that expression of SMAD4 and E-cadherin in the TGF-β/SMAD signaling pathway was under direct miR-362 negative regulation to suppress cell migration and invasion *in vitro* and metastasis* in vivo*, as summarized in the scheme presented in Figure 6A*.* On the other hand, our data (Fig. 1) showed that miR-362 did not seem to directly target SNAI1; the SNAI1 results observed here were likely regulatory consequences by SMAD4. Our findings are consistent with other reports that negative regulation of *SMAD4* expression by other miRNAs is associated with suppressed cell motility and invasiveness in different cancers 31-34. Clinically, SMAD4 has been linked with metastasis in a number of cancers 31, 32, 34-37. In colorectal cancer, SMAD4 has also been previously shown to accumulate in the nucleus in the EMT process and is associated with lymphatic invasion 31.

An observed anomaly in miR-362 regulation of SMAD4 expression in this work is that sponge-induced miR-362 knockdown also down-regulated SMAD4 expression both *in vitro* and *in vivo* (Figs. 1C - 1F & 4), albeit at a lower extent than the SMAD4 down-regulation on miR-362 over-expression. The data clearly suggested that miR-362 also positively regulate SMAD4 expression. Functionally, miR-362 knockdown-induced SMAD4 down-regulation was also associated with suppressed cell motility and invasiveness *in vivo* (Figs. 4 & 5, Table 1). This anomaly may be explained by the existence of miR-362-targeted SMAD4 suppressors which, when negatively regulated by miR-362, results in down-regulated SMAD4 levels. Two such SMAD4 suppressors, BRK and DACH1, are shown in Figure 6B to illustrate the proposed positive miR-362 regulation of SMAD4 expression. BRK (breast tumor kinase), also called tyrosine-protein kinase 6 (*PTK6*), is a nonreceptor protein tyrosine kinase that is highly expressed in breast cancer cells 38. Importantly, BRK has been shown to phosphorylate SMAD4, destinating SMAD4 to ubiquitin-mediated proteasomal degradation 39. On bioinformatics analysis, a miR-362-5p target site is found in the 3'UTR of the BRK transcript (Fig. 6B and Suppl. Fig. S2). Hence, miR-362-3p targeting of BRK, a SMAD4 repressor, would have led to down-regulated SMAD4 expression (Fig. 6B). A second SMAD4 repressor DACH1 (Dachshund homolog 1) is a tumor suppressor of malignant proliferation 40-43 and suppresses cell migration and invasion 44, 45. Relevant to this work is the report and DACH1 inhibits TGF-β signaling through binding with SMAD4 46. Bioinformatics analysis has also predicted at least seven putative miR-362-5p and -3p target sites in the 3'UTR of the *DACH1* mRNA (see Suppl. Fig. S2). As an example (Fig. 6B), miR-362 knockdown would have up-regulated DACH1 expression to deplete SMAD4, through heteromeric binding, from being available for formation of phosphorylated-SMAD2/3-SMAD4 complex for subsequent transcriptional activation in the nucleus.

There are, indeed, clinical reports that miRNA knockdown in some cancers resulted in suppressed migration and invasion via SMAD4 regulation 6, 47, 48. As an example, SMAD4 suppression by miR-144 inhibited cellular migration and invasion and suppressed metastasis 32 as shown in this work. Furthermore, there are other precedents of miRNA positive regulation of the EMT process via feedback, double feedback or forward-feed loops between miRNAs and the targets, such as SNAI1 and E-cadherin (reviewed in 26, 27). Zhang et al. (2019) 49 have recently shown that the SMAD2/3 complex binds to the promoter of the miR-362 gene to positively regulate miR-362 expression (Fig. 6A, blue lines). Since SMAD2/3 expression is itself under TGF-β regulation, a miR-362 feedforward loop of regulation may have been formed. In summary, miR-362 regulation of SMAD4 expression, and, therefore, the TGF-β/SMAD signaling, is a collective consequence of both negative and positive regulation of SMAD4 expression, resulting in repressed cellular migration and invasion *in vitro* and metastasis *in vivo* on miR-362 knockdown. In summary, miR-362 regulates the TGF-β/SMAD signaling pathway both negatively and positively via a multi-thronged regulation of the SMAD proteins, in particular SMAD4.

## Conclusion

Data of this work indicate that miR-362 is an integral part of the TGF-β/SMAD signaling pathway, and that miR-362 is the driver of a previously-unreported SMAD4/E-cadherin/miR-362 axis in modulating the EMT and MET processes, and, therefore, cell motility and invasiveness in cancers, superseding other reported miR-362 targets in explaining miR-362 suppression of metastasis 8, 12, 13, 16, 19-21. Anti-miR-362 strategies, using appropriate inhibitors, such as the miR-362 sponges described here, may be tested for therapeutic suppression of cancer cell metastasis in miR-362-positive cancers.

## Supplementary Material

Supplementary figures and tables.

## Figures and Tables

**Table 1:** Suppressed tumor growth and metastasis in miR-362-knockdown orthotopic mice

Mouse	Tumor volume (mm^3^)	Metastatic loci
Negative control		
#27	2,560	2
#28	2,688	3
#76	900	2
#78	1,690	None
miR-362-3p sponge		
#31	None	None
#83	None	None
#85	<50	None
#86	None	None
miR-362-5p sponge		
#29	None	None
#30	<50	None
#79	None	None
#80	<50	None

Nude mice were injected orthotopically with different batches of miR-362 sponge-transduced cells (n=6). Only mice that survived 80 days or more post-cell injection were subjected to further analysis. Negative control (NC) mice were injected with cells transduced with a blank sponge vector.

## Abbreviations

CDH1: E-cadherin
EMT: epithelial-to-mesenchymal transition
GFP: green fluorescence protein
MET: mesenchymal-to-epithelial transition
PI3K-Akt: phosphatidylinositol-3-kinases-protein kinase B
TGF-β: transforming growth factor-beta
3'UTR: 3'untranslated region
