# Supplementary material for: MicroRNA-362 negatively and positively regulates SMAD4 expression in TGF-β/SMAD signaling to suppress cell migration and invasion
Source: Int J Med Sci. 2021 Feb 18;18(8):1798–809. doi: 10.7150/ijms.50871 (PMC7976584; doi:10.7150/ijms.50871)

Supplementary Table S1. Sequences of primers used in constructing luciferase plasmids and site-directed mutagenesis of the miRNA target sequences

**Gene**<br>(miR362 target sites)	**Wild-type construct**	**Mutant construct**
**SMAD4**<br>(containing both the miR362-5p & -3p sites)	**SMAD4-SacI-Forward primer**<br>5′-AGT**GAGCTC**GGGACTTCCCCATGGACATT-3′<br><br>**SMAD4-XhoI-Reverse primer**<br>5′- AGT**CTCGAG**ACCATCAAACCAGGCACAGA-3′	**SMAD4-Mut1 (miR362-5p)**<br>**First set:**<br>**Luciferase-SacI-Forward primer**<br>5′-CTAGTTGTTTAAAC**GAGCTC**-3′<br><br>**SMAD4-Mutant-Reverse primer**<br>5′-GAAAGATA***taggagt***ATTTTATTTCATACTATTTATCCTG-3′<br><br>**Second set:**<br>**SMAD4-Mutant-forward primer**<br>5′-GAAATAAAAT***actccta***TATCTTTCAGATGTGTTTACTTTTG-3′<br><br>**SMAD4-XmaI-Reverse primer**<br>5′-AATCAGTTGAA**CCCGGG**AG-3′<br><br>**SMAD4-Mut2 (miR362-3p)**<br>**First set:**<br>**SMAD4-BstZ17I-Forward primer**<br>5′-GCCCCTG**GTATAC**AAAGATAATGACAATA-3′<br><br>**SMAD4-Mutant-Reverse primer**<br>5′-ACACCCCCCTTT***cacacaa***ACACCCTGCCC-3′

		**Second set:**<br>**SMAD4-Mutant-Forward primer**<br>5′-GGCAGGGTGT***ttgtgtg***AAAGGGGGGTGTTTG-3′<br><br>**SMAD4-XhoI-Reverse primer**<br>5′-TAGA**CTCGAG**ACCATCAAACCAGGCA-3′
**SNAI1**<br>(miR-362-5p site)	**SNAI1-SacI-Forward primer**<br>5′- GCT**GAGCTC**CCCTCGAGGCTCCCTCTT-3′<br><br>**SNAI1-XbaI-Reverse primer**<br>5′-GCA**TCTAGA**TGCTTTATTGAATATCAATAAAC-3′	**SNAI1-Mut**<br>**First set:**<br>**XmaI SNAI1-Forward primer**<br>5′-GTGGCACCTGTTT**CCCGGG**CAATTTAAC-3′<br><br>**SNAI1-Reverse primer**<br>5′-CTATACAAAACGTT***aggagt***ATACAAAATGTTTGAAATATAAATACC-3′<br><br>**Second set:**<br>**XmaI SNAI1-Forward primer**<br>5′-GTGGCACCTGTTT**CCCGGG**CAATTTAAC-3′<br><br>**SNAI1-Reverse primer**<br>5′-TGCCTGCAGGTCGAC**TCTAGA**TGCTTTATTGAATATCAATAAACTGTACATATAACTATACAAAACGTT***aggagt***ATAC-3′
CDH1<br>(Two miR-362-3p sites)	**CDH1-SacI-Forward primer**<br>5′- ATC**GAGCTC**CCCAGCACCTTGCAGATTTTC-3′<br><br>**CDH1-XbaI-Reverse primer**<br>5′-ATC**TCTAGA**TTCCGCTCTGTCTTTGGCTG-3′	**CDH1-Mut1**<br>**First set:**<br>**Luciferase-SacI-Forward primer**<br>5′-CTAGTTGTTTAAAC**GAGCTC**-3′<br><br>**CDH1-Mutant-Reverse primer:**<br>5′-AGAGGG***cacacaa***AGTGTAGTAATGAGCAGAAG-3′

		**Second set:**<br>**CDH1-mutant-Forward primer**<br>5′-CTACACT***ttgtgtg***CCCTCTGCCTTTTTTTTTTTTTAAG-3′<br><br>**CDH1-EcoRI-Reverse primer**<br>5′-GCAAAGCAACT**GAATTC**AGG-3′<br><br>**CDH1-Mut2**<br>**First set:**<br>**CDH1-EcoRI-Forward primer**<br>5′-TCAACTCTCACTCCT**GAATTC**-3′<br><br>**CDH1-mutant-Reverse primer**<br>5′-CATTAATG***ccacaca***TTACTCAGAACAAGTCACTG-3′<br><br>**Second set:**<br>**CDH1-mutant-Forward primer**<br>5′-TGAGTAA***tgtgtgg***CATTAATGTTTATTTAGCTCTG-3′<br><br>**CDH1-XbaI-Reverse primer**<br>5′-TGCAGGTCGAC**TCTAGA**TTC-3′

Letters in bold uppercase letters and underlined are restriction enzyme recognition sequences. Letters in bold lowercase italics letters and underlined are mutated seed sequences.

Supplementary Figure S1

Analysis of miR-362-5p/-3p expression levels from subcutaneous tumors with miR-362-5p/-3p knockdown (k/d). Mice were generated using different cell batches and were designated different mouse numbers.

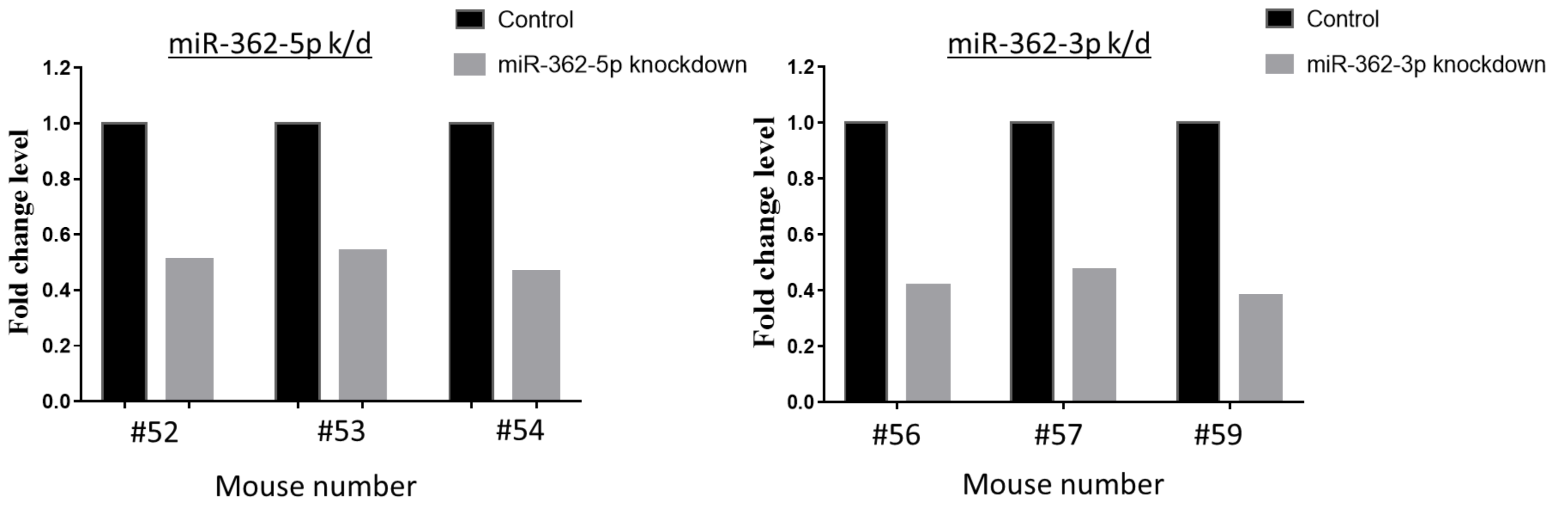

Supplementary Figure S2

Putative miR-362-3p/-5p binding sites in 3'UTR of the DACH1 and BRK transcripts

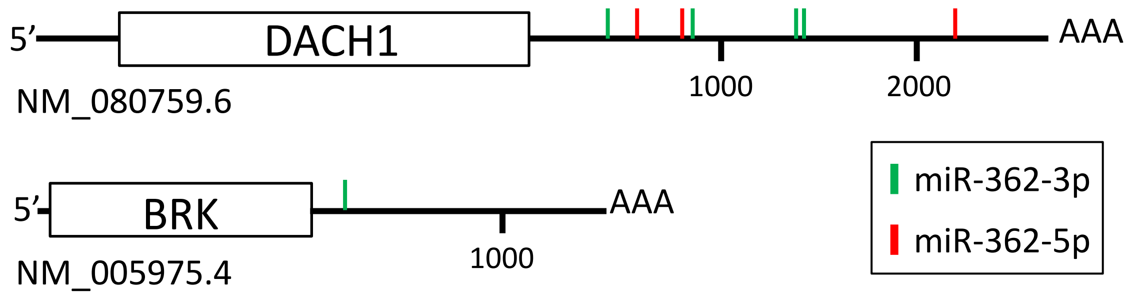

Supplement: Supplementary file 1 — Supplementary figures and tables. [file ijmsv18p1798s1.pdf]
